# Primary Intranodal Epithelioid Haemangioendothelioma in the Submandibular Region: A Case Report

**DOI:** 10.1007/s12070-024-04752-2

**Published:** 2024-06-06

**Authors:** Khilan Shukla, Touraj Taheri, Hooman Baghaie, Omar Breik

**Affiliations:** 1 Maxillofacial Surgery Department, Royal Brisbane and Women’s Hospital, Brisbane, Australia; 2 Griffith University, Gold Coast, Australia; 3 James Cook University, Cairns, Australia; 4 Pathology Department, Royal Brisbane and Women’s Hospital, Brisbane, Australia; 5 School of Medicine, University of Queensland, Brisbane, Australia; 6 Maxillofacial Surgery Department, Westmead Hospital, Sydney, Australia

**Keywords:** Epithelioid haemangioendothelioma, Submandibular gland, Malignancy, Head and neck surgery, Head and neck pathology

## Abstract

Epithelioid haemangioendothelioma (EHE) is a rare vascular tumour that primarily affects the liver, lungs and bone. It is very rarely described in the head and neck region, and is exceptionally uncommon within the submandibular region. We report a very rare case of EHE originating in a lymph node within the submandibular salivary gland of a 54-year-old female patient. The tumour was resected and the patient was regularly followed up, with no recurrence of disease at 24 months postoperatively. A review of existing literature, clinical and immunohistopathological features are discussed, which highlight the diagnostic dilemma, absence of consensus for management and appropriate surveillance method associated with EHE.

## Introduction

Epithelioid haemangioendotheliomas (EHE) are a group of rare vascular tumours that primarily affect the liver, lungs and bones, with very rare incidence in the heart, spleen, breast, and in the head and neck [1]. After having been previously termed intravascular bronchoalveolar tumour and histiocytoid haemangioma [2], the term ‘*Epithelioid haemangioendothelioma’* was first definitively described in 1982 by Weiss and Enzinger [3], which allowed for its differentiation from other tumours of endothelial origin. EHE was initially considered a clinically intermediate condition between angiosarcoma and haemangioma, however is now recognised as distinct from these two conditions after identification of disease defining genes [4].

Nevertheless, due to its rarity and varied clinical and histological presentation, it is still commonly misdiagnosed [5, 6]. Several papers report EHE as having been initially misdiagnosed as epithelioid haemangioma, angiosarcoma, hepatocellular carcinoma, and sarcoma [7, 8], with one study noting as many as 60–80% of hepatic EHE being initially misdiagnosed [8].

The presence of EHE in the head and neck is considered very rare, however, given the propensity for misdiagnosis, it is possible that it is underreported in the literature. After systematically reviewing the English literature there are only eight cases [9–13] of EHE reported within the submandibular region since Weiss and Enzinger [14] defined the disease in 1982 (Table 1). It is an uncommon differential of a lump in the submandibular region, but should still be clinically and histologically excluded during the diagnostic process. We report a rare case of primary intranodal EHE in the right submandibular gland, which was managed surgically and had no evidence of clinical or radiological recurrence at 24 months.

**Table 1:** Reports of EHE in the submandibular region

Study (Year)	Case(s)	Age + Sex (M/F)	Primary (*P*) vs. Metastatic (M)	Treatment	Follow up	Outcome
Ellis & Kratochvil (1986)^9^	4	54 (F)	P	Surgical Excision	9 months	No recurrence
		50 (M)	P	Surgical Excision	N/R	N/R
		4 (F)	Unknown	Surgical Excision	N/R	N/R
		67 (M)	P	Surgical Excision	N/R	Residual tumour excised at 2 months
Naqvi et al. (2008)^10^	1	62 (F)	N/A	N/A	N/R	N/R
Ranjit et al. (2015)^11^	1	25 (F)	P	Surgical Excision	N/R	N/R
Rigby et al. (2006)^12^	1	N/A	P	N/A	N/R	N/R
Yoruk et al. (2008)^13^	1	44 (F)	P	Surgical Excision	6 months	No recurrence

N/A: Not Available

N/R: Not Reported

## Case Report

A 58-year-old woman was referred to our oral and maxillofacial surgery clinic with a 4-month history of a painful submandibular lump. The lump had not changed in size and she denied any dysphagia, odynophagia or odontogenic pain and did not report fevers, chills, or night sweats. She was otherwise in good health and did not smoke or drink alcohol. She reported removal of a benign parathyroid adenoma 11 years ago.

On examination, a firm 2 cm x 1 cm tender, non-mobile mass was noted in the right submandibular region. There was no other cervicofacial lymphadenopathy, purulent intraoral discharge or any overlying cutaneous features of cellulitis. Her dentition was sound, and a dental radiograph did not reveal an odontogenic source for her presentation. A prior ultrasound examination ordered by her general practitioner revealed inflammation around the right submandibular gland, with no evidence of salivary calculi.

**Fig. 1:**
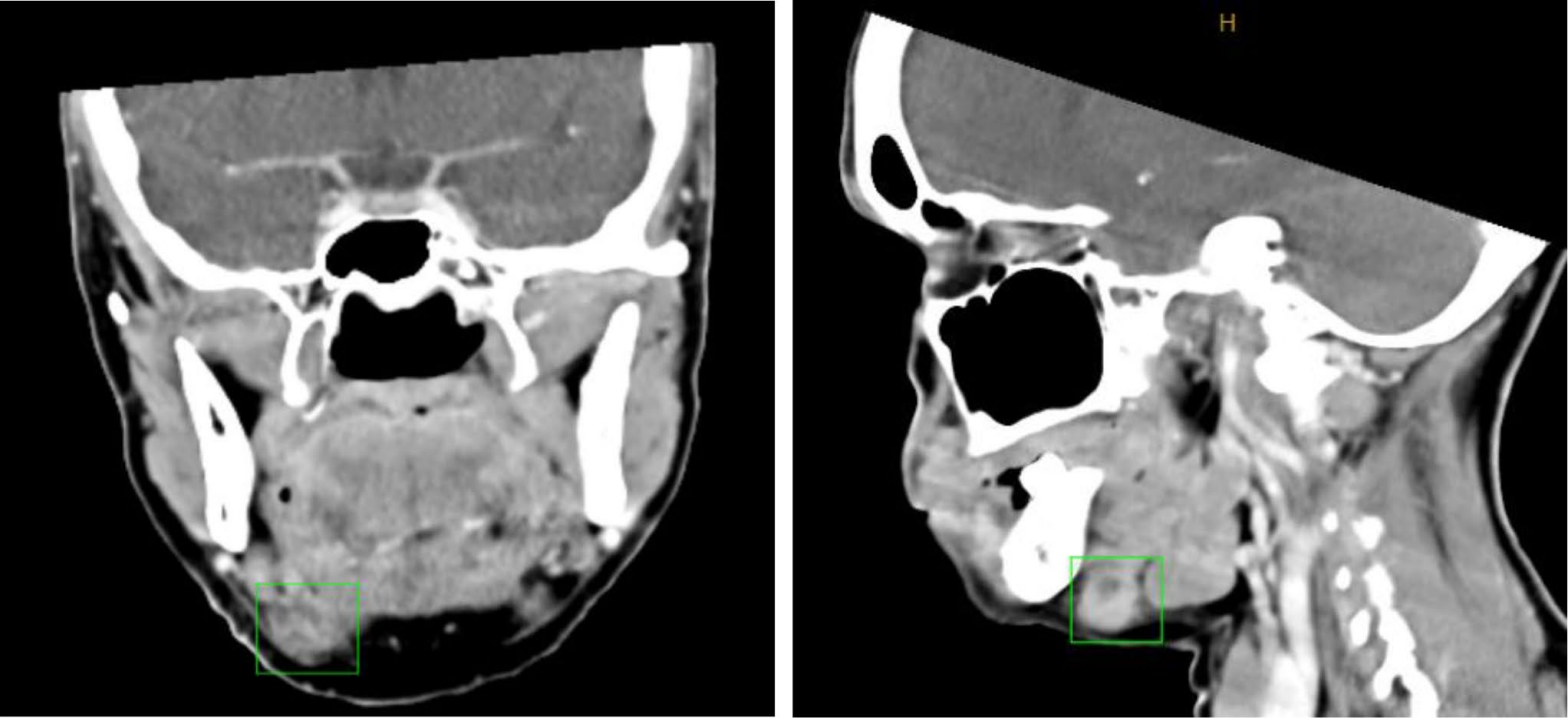
CT scan demonstrating enlarged right submandibular lymph node with necrotic appearance

A IV contrast-enhanced computed tomography (CT) of her neck demonstrated an enlarged right submandibular lymph node, with central hypodensity and peripheral fat stranding, suggesting the presence of a necrotic node (Fig. 1). Fine needle aspiration biopsy (FNAB) was inconclusive with a mixed population of lymphoid cells in a background of blood. Follow up flow cytometry revealed no malignant cells.

**Fig. 2:**
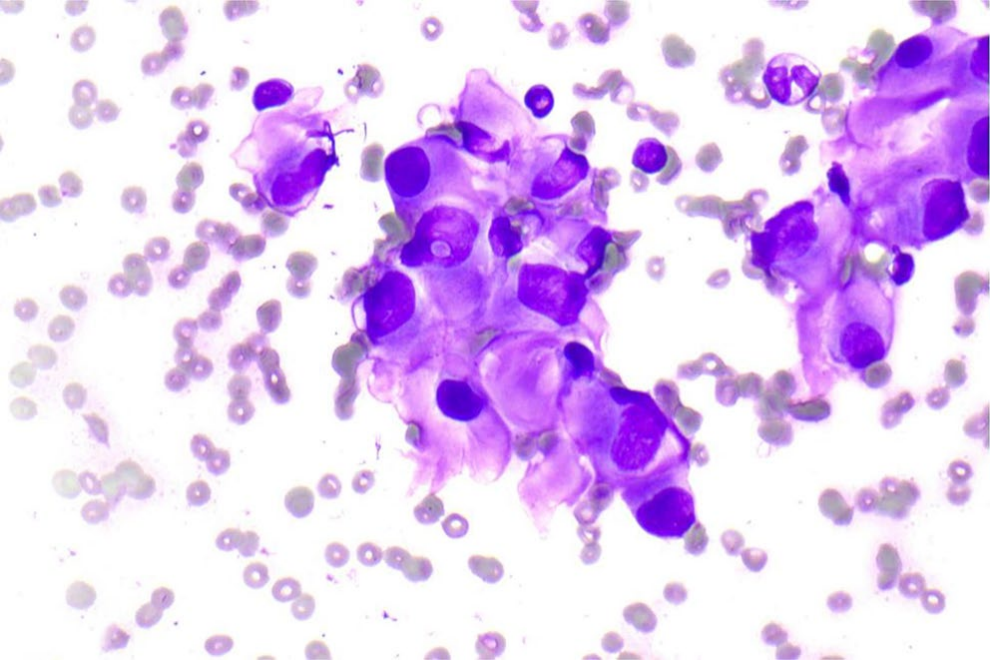
Cytology demonstrating mild atypia of epithelioid and plasmacytoid cells. No mitosis or high grade atypia evident

Given the unusual and prolonged nature of the patient’s presentation, there was a high degree of suspicion for a neoplastic process. A repeat FNAB was consequently completed which noted presence of epithelioid cells with low grade morphological features (Fig. 2).

**Fig. 3:**
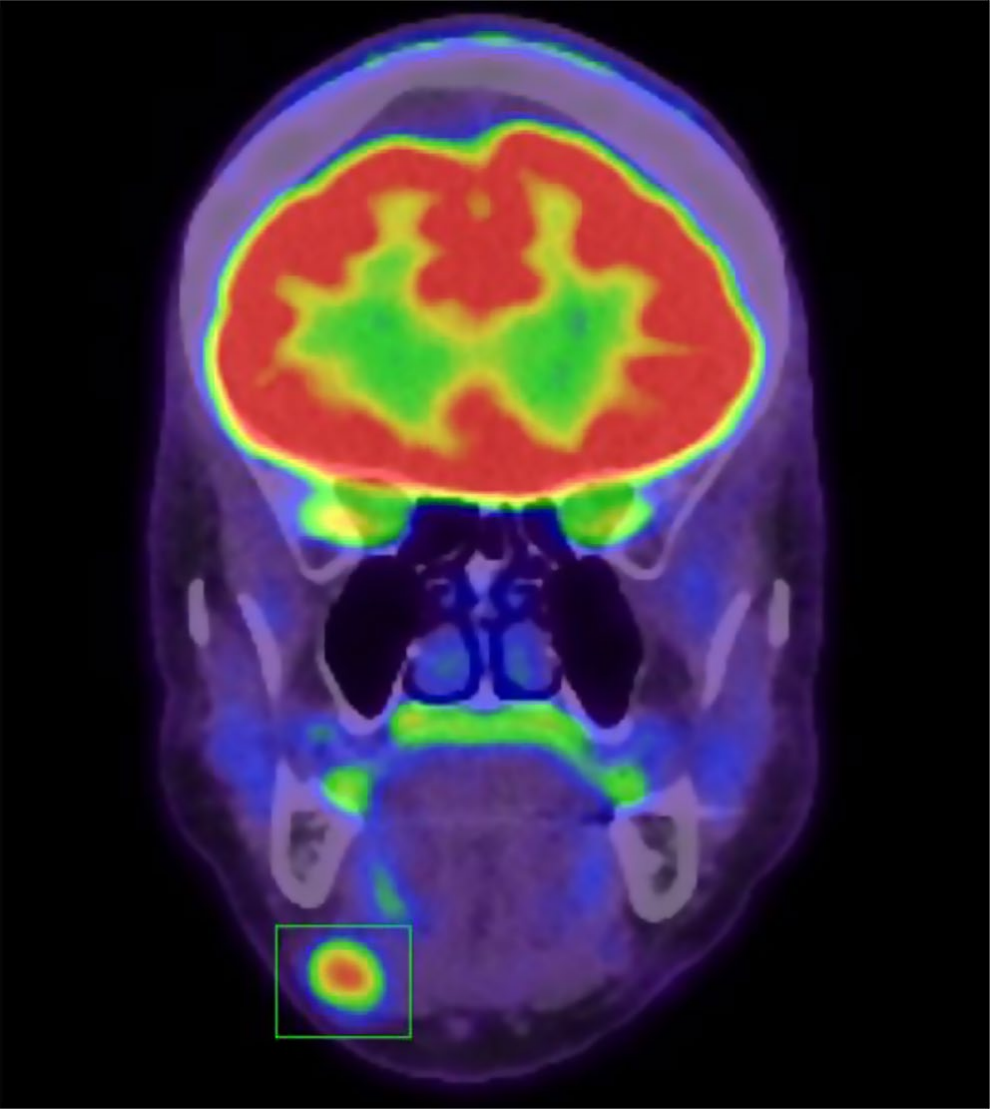
FDG-PET scan demonstrating moderately avid lesion in right submandibular region

A fluorodeoxyglucose positron emission tomography (FDG-PET) scan demonstrated moderately avidity (Fig. 3) of a primary intranodal tumour in the right submandibular gland, with no evidence of further nodal or metastatic disease. There was no tumour located within the salivary parenchyma. The possibility of a primary thyroid neoplasm with lymph node metastasis was considered, however a subsequent ultrasound guided core biopsy revealed features consistent with EHE.

**Fig. 4:**
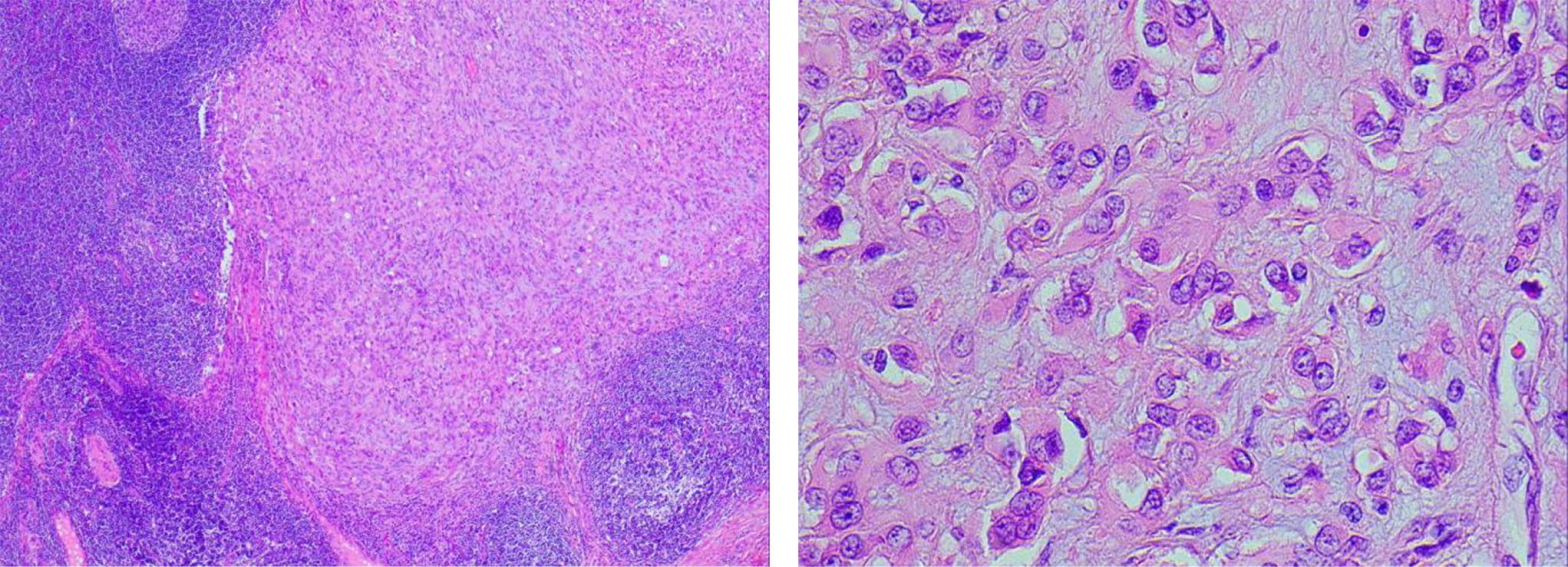
Histopathology slides demonstrating cords or nests of epithelioid cells with cytoplasmic vacuolization and myxohyaline stroma. High power view (right) demonstrated abundant eosinophilic cytoplasm and intracytoplasmic vacuoles with pseudo-lumen formation

The core biopsy histology revealed classic features of epithelioid hemangioendothelioma presenting as an intranodal tumour. The tumour cells were round and epithelioid with mild nuclear pleomorphism, nil mitosis or necrosis and abundant eosinophilic cytoplasm. Some intracytoplasmic vacuoles with pseudo-lumen formation were noted. A myxoid stroma with hyalin change and small capillary channels was noted. The tumour cells were decorated by endothelial markers CD31, CD34 and ERG. Cytokeratin AE1/AE3 and S100 were negative. Histologic features from the resected specimen are demonstrated in Fig. 4.

After a multidisciplinary team discussion and review of her imaging and histopathology, the patient was treated by a right sided neck dissection of Level I to III nodes. The operative specimen histology yielded fourteen nodes with an isolated pathological node with focal extracapsular extension in Level IB. The Level II and III nodes were free of disease. Her recovery was unremarkable, and she was followed up clinically every 6 months, with biannual FDG-PET scans. She did not receive any adjuvant chemotherapy or radiotherapy and there was no clinical or radiographic evidence of recurrence at 24 months to date.

## Discussion

Within the head and neck, haemangioendotheliomas account for fewer than 1% of vascular tumours. Four major types have been described - epithelioid, hobnail, polymorphous and kaposiform [13], with the epithelioid having the poorest prognosis due to its aggressiveness and potential for metastasis [15]. Recent advancements in genotyping have allowed for identification of disease defining gene fusions such as WWTRI/CAMTA1 and YAP1/TFE3 using fluorescence *in* situ hybridisation (FISH) [16, 17]. These establish EHE as a distinct entity and allow for its immunohistological differentiation from similar benign and malignant tumours. In our case, FISH for CAMTA1 could not be sourced, however this is not mandatory for diagnosis if other histological features suggest EHE [18, 19].

This case report detailed a clearly defined EHE originating in a lymph node within the submandibular gland. The reported age of incidence of EHE in the submandibular region ranges between 4 and 67 years, however appears to have a predilection for middle and older aged adults. The aetiology of this condition is still largely unknown [20]. The patient in our case was 58 years old, which is consistent with the majority of presentations.

Epithelioid haemangioendothelioma was reclassified from ‘intermediate malignancy’ to ‘fully malignant’ by the World Health Organisation in 2013 [21]. Due to its rarity, a standardised treatment protocol is still lacking for EHE, with several proposed treatment regimens based on the location and nature of the tumour. Localised lesions are typically treated with surgery, although chemotherapy and radiotherapy may also be used to reasonable effect. Alternate regimens that are reported in the literature include hormone therapy, thermoablation and transarterial chemoembolisation, however these are not well described or widely used [22, 23]. Historically, some authors utilised a ‘watchful waiting’ approach, with spontaneous regression and prolonged disease stability having been reported in very rare instances [24, 25]. As the tumour is now classified as ‘fully malignant’ and given the elevated risk of metastasis from head and neck tumours, treatment with curative intent is usually indicated, and active surveillance should only be considered for patients who are poor candidates for surgery or active treatment [26].

To date, there is a severe paucity of evidence to for a standardised approach to managing EHE in the head and neck. A recent consensus paper by Stacchiotti et al. (2021) [26] notes that surgery with curative intent is usually appropriate in cases of unifocal EHE. In the 8 available cases of EHE reported in the submandibular region (Table 1), 6 patients underwent surgical treatment with wide local excision [9, 11, 13], with insufficient data for the remaining 2 papers [10, 12]. None of these papers clearly described the location of the tumour as originating within a submandibular lymph node, however Yoruk noted presence of lymphoid tissue on FNAB [13]. Ellis and Kratochvil (1986) reported a patient case where residual EHE tumour required removal after 2 months. They also noted a second case of EHE in the submandibular region, with a similar tumour in the alveolar mucosa [9]. It was unable to be determined which specimen represented the primary tumour. Therefore, it is important to conduct an appropriate clinical examination and investigations to rule out the possibility of a distant primary sites with metastasis to the head and neck, as metastatic lesions may be clinically and histologically indistinct from a primary tumour. In our patient, there was no additional tumour found on FDG-PET.

Unfortunately, the follow up of submandibular EHE was sparsely reported, and was six and nine months in the 2 patients where data was available. There remains a lack of available data regarding optimal frequency and follow up duration of EHE, however Stacchiotti et al. [26] suggest MRI of the primary site and whole-body CT scan 6-monthly for the first 5 years. They note that avidity on FDG-PET scans are usually mild-moderate, with increased avidity being proportional to the risk of progression and poorer survival [26]. In the current patient, as the initial lesion demonstrated avidity, and given the lack of an established screening protocol at the time of treatment, the patient was followed up with 6-monthly surveillance FDG-PET and did not display signs of recurrence at 24 months follow up. Original and subsequent imaging studies including CT scan and PET did not show a primary or secondary tumour in lung, liver, soft tissue or bone. Therefore this case can be interpreted as primary intranodal EHE in the right submandibular gland which has only been reported once before in the literature [13, 27].

## Conclusion

Epithelioid haemangioendothelioma remains an uncommon presentation in the head and neck, and is especially rare in the submandibular region. Nonetheless, adequate investigation should be undertaken to exclude this condition during the diagnostic process of a submandibular lump. If diagnosed, appropriate investigation should be undertaken to confirm whether it is a primary or metastatic lesion. Due to its rarity in the submandibular region, even if a lesion is presumed to be a primary tumour, surveillance may be required to ensure it is not a metastatic lesion. While the majority of localised EHE tumours in the head and neck are usually managed surgically, further research is required regarding the optimal imaging modality and surveillance of these lesions post-operatively. The follow up and monitoring of primary and metastatic EHE lesions in the head and neck would benefit from further investigation.
